# Supplementary material for: Heat adaptation of phage T7 under an extended genetic code
Source: Virus Evol. 2021 Dec 1;7(2):veab100. doi: 10.1093/ve/veab100 (PMC8923235; doi:10.1093/ve/veab100)
Supplement: veab100_Supp [file veab100_supp.zip › Sequencing_Analysis.zip!/Sequencing_Analysis/A3/evidence/JC_SIDE_2_75.html]

BRESEQ :: Evidence


| New junction evidence | | | | | | | | | | | |
| --- | --- | --- | --- | --- | --- | --- | --- | --- | --- | --- | --- |
|  | | seq id | position | reads (cov) | reads (cov) | score | skew | freq | annotation | gene | product |
| \* | ? | NC\_001604.1 | = 19635 | 393 (0.230) | 1117 (0.650) | 309/590 | 0.2 | 74.6% | intergenic (–/–) | *–/–* | –/– |
|  | ? | NC\_001604.1 | 19645 = | 374 (0.220) |  |  |  |  | intergenic (–/–) | *–/–* | –/– |

Only 100 of 1528 total aligned reads displayed.  

`ATTCACCACAAAGGAGACATTCTGGATGTAACGTCCGTTGTCATTAATCCTGAGGCAGACTCTAAGGGCTTACAGCGATTCCTAGCGAAACGCTTTAAGTACCTTGCGGAACTCCACGATTGCGATTGGGTGTCTCGTTGTAAGCATGAAGGCGAGACAATGCGTGTATACTTTAAGGAGGTATAAGTTATGGGTAAGAAAGTTAAGAAGGCCGTGAAGAAAGTCACCAAGTCCGTTAAGAAAGTCGTTAAGGAAGGGGCTCGTCCGGTTAAACAGGTTGCTGGCGGTCTAGCTGGTCTGGCTGGTGGTACTGGTGAAGCACAGATGGTGGAAGTACCACAAGCTGCCGCACAGATTGTTGACGTACCTGAGAAAGAGGTTTCCACTGAGGACGAAGCACAGACAGAAAGCGGACGCAAGAAAGCTCGTGCTGGCGGTAAGAAATCCTTGAGTGTAGCCCGTAGCTCCGGTGGCGGTATCAACATTTAATCAGGAGGTTATCGTGGAAGACTGCATTGAATGGACCGGAGGTGTCAACTCTAAGGGTTATGGTCGTAAGTGGGTTAATGGTAAACTTGTGACTCCACATAGGCACATCTA  >  NC_001604.1/19346‑19945`  

                                                                                                                                                                                                                                                                                                           `|`                                                                                                                                                                                                                                                                                                              
  `tataagagcccgccacaaaggagacattctggatgtaacttccgttggcattaatcctgaggcagactctaagggcttacagcgattcctagcgaaacgctttaagtaccttgcggaactccacgattgcgattgggtgtctcgttgtaagcatgaaggcgagacaatgcgtgtatactttaaggaggtataagttatgggtaagaaagttaagaaggccgtgaagaaagtcacaaagtccgttaagaaagtcgttaaggaaggggctcgtccggttaaacaggttgctggcggtctggct                                                                                                                                                                                                                                                                                                           <  1:8332‑M2/4‑1 (MQ=255)`  
  `ctgtgacattcaccacaaaggagacattctggatgtaacgtccgttgtcattaatcctgaggcagactctaagggcttacagcgattcctagcgaaacgctttaagtaccttgcggaactccacgattgcgattgggtgtctcgttgtaagcatgaaggcgagacaattcgtgtatactttaaggaggtataagttatgggtaagaaagttaagaaggccgtgaagaaagtcaccaagtccgttaagaaagtcgttaaggaaggggctcgtccggttaaacaggttgctggcggtctggct                                                                                                                                                                                                                                                                                                           >  1:45686‑M2/298‑301 (MQ=255)`  
         `attcaccacaaaggagacattctggatgtaacgtccgttgtcattaatcctgaggcagactctaagggcttacagcgattcctagcgaaacgctttaagtaccttgcggaactccacgattgcgattgggtgtctcgttgtaagcatgaaggcgagacaatgcgtgtatactttaaggaggtataagttatgggtaagaaagttaagaagtccgtgaagaaagtcaccaagtccgttaagaaagtcgttaaggaaggggctcgtccggttaaacaggttgctggcggtctGGCTGGTGGTa                                                                                                                                                                                                                                                                                                    >  1:255170‑M2/291‑301 (MQ=255)`  
         `attcaccacaaaggagacattctggatgtaacgtccgttgtcattaatcctgaggcagactctaagggcttacagcgattcctagcgaaacgctttaagtaccttgcggaactccacgattgcgattgggtgtctcgttgtaagcatgaaggcgagacaatgcgtgtatactttaaggaggtataagttatgggtaagaaagttaagaaggccgtgaagaaagtcaccaagtccgttaagaaagtcgttaaggaaggggctcgtccggttaaacaggttgctggcggtctGGCTGGTGGTa                                                                                                                                                                                                                                                                                                    >  1:130419‑M2/291‑301 (MQ=255)`  
            `ggAGACATTCTGGATGTAACGTCCGTTGTCATTAATCCTGAGGCAGACTCTAAGGGCTTACAGCGATTCCTAGCGAAACGCTTTAAGTACCTTGCGGAACTCCACGATTGCGATTGGGTGTCTCGTTGTAAGCATGAAGGCGAGACAATGCGTGTATACTTTAAGGAGGTATAAGTTATGGGTAAGAAAGTTAAGAAGGCCGTGAAGAAAGTCACCAAGTCCGTTAAGAAAGTCGTTAAGGAAGGGGCTCGTCCGGTTAAACAGGTTTCTGGCGGTCTAGCTGGTCTGGCtggtggctgtt                                                                                                                                                                                                                                                                                                 >  1:5196/1‑296 (MQ=255)`  
            `caccacaaaggagacattctggatgtaacgtccgttgtcattaatactgaggcagactctaagggcttacagcgattcctagcgaaacgctttaagtaccttgcggaactccacgattgcgattgggtgtctcgttgtaagcatgaaggcgagacaatgcgtgtatactttaaggaggtataagttatgggtaagaaagttaagaaggccgtgaagaaagtcaccaagtccgttaagaaagtcgttaaggaaggggctcgtccggttaaacaggttgctggcggtctGGCTGGTGGTACTg                                                                                                                                                                                                                                                                                                 >  1:71475‑M2/288‑301 (MQ=255)`  
              `ccacaaaggagacattctggatgtaacgtccgttgtcattaatcctgaggcagactctaagggcttacagcgattcctagcgaaacgctttaagtaccttgcggaactccacgattgcgattgggtgtctcgttgtaagcatgaaggcgagacaatgcgtgtatactttaaggaggtataagttatgggtaagaaagttaagaaggccgtgaagaaagtcaccaagtccgttaagaaagtcgttaaggaaggggctcgtccggttaaacaggttgctggcggtctGGCTGGTGGTACtggc                                                                                                                                                                                                                                                                                               >  1:218906‑M2/286‑300 (MQ=255)`  
                 `caaaggagacattctggatgtaacgtccgttgtcattaatcctgaggcagactctaagggcttacagcgattcctagcgaaacgctttaagtaccttgcggaactccacgattgcgattgggtgtctcgttgtaagcatgaaggcgagacaatgcgtgtatactttaaggaggtataagttatgggtaagaaagttaagaaggccgtgaagaaagtcaccaagtccgttaagaaagtcgttaaggaaggggctcgtccggttaaacaggttgctggcggtctGGCTGGTGGTACTGGTGaa                                                                                                                                                                                                                                                                                            >  1:154356‑M2/283‑301 (MQ=255)`  
                 `caaaggagacattctggatgtaacgtccgttgtcattaatcctgaggcagactctaagggcttacagcgattcctagcgaaacgctttaagtaccttgcggaactccacgattgcgattgggtgtctcgttgtaagcatgaaggcgagacaatgcgtgtatactttaaggaggtataagttatgggtaagaaagttaagaaggccgtgaagaaagtcaccaagtccgttaagaaagtcgttaaggaaggggctcgtccgggtaaacaggttgctggcggtctGGCTGGGGGTACTGGTGaa                                                                                                                                                                                                                                                                                            >  1:200850‑M2/283‑301 (MQ=255)`  
                     `ggagacattctggatgtaacgtccgttgtcattaatcctgaggcagactctaagggcttacagcgattcctagcgaaacgctttaagtaccttgcggaactccacgattgcgattgggtgtctcgttgtaagcatgaaggcgagacaatgcgtgtatactttaaggaggtataagttatgggtaagaaagttaagaaggccgtgaagaaagtcaccaagtccgttaagaaagtcgttaaggaaggggctcgtccggttaagcaggttgctggcggtctGGCTGGTGGTCCTGGTGAAGcac                                                                                                                                                                                                                                                                                        >  1:326113‑M2/279‑301 (MQ=255)`  
                       `ggATGTAACGTCCGTTGTCATTAATCCTGAGGCAGACGCTAAGGGCTTACAGCGATTCCTAGCGAAACGCTTTAAGTACCTTGCGGAACTTCACGATTGCGATTGGGTGTCTCGTTGTAAGCATGACGGCGAGACAATGCGTGTATACTTTAAGGAGGTATAAGTTATGGGTAAGAAAGTTAAGAAGGCCGTGAAGAAAGTCACCAAGTCCGTTACGAGAGTCGTTAAGGAAGGGGCTCGTCGGGTTAAATAGGTTTCTGGCGGTCTAGCTGGTCTGGCTGGTGGTACTGGTGAAGCACAg                                                                                                                                                                                                                                                                                      >  1:334831/1‑301 (MQ=255)`  
                       `agacattctggatgtaacgtccgttgtcattaatcctgaggcagactctaagggcttacagcgattcctagcgaaacgctttaagtaccttgcggaactccacgattgcgattgggtgtctcgttgtaagcatgaaggcgagacaatgcgtgtatactttaaggaggtataagttatgggtaagaaagttaagaaggccgtgaagaaagtcaccaagtccgttaagaaagtcgttaaggaaggggctcgtccggttaaacaggttgctggcggtctGGCTGGTGGTACTGGTGAAGCACAg                                                                                                                                                                                                                                                                                      >  1:203758‑M2/277‑301 (MQ=255)`  
                         `ttgtataagagccagcgtccgttgtcgttaatcctgaggcagactctaagggcttacagcgattcctagcgaaacgctttaagtaccttgctgaactccacgattgcgattgggtgtctcgttgtaagcatgaaggcgagacaatgcgtgtatactttaaggaggtataagttatgggtaagaaagttaagaaggccgtgaagaaagtcaccaagtccgttaagaaagtcgttaaggaaggggctcgtccggttaaacaggttgctggcggtctGGCTGGTGGTACTGGTGAAGCACAGAt                                                                                                                                                                                                                                                                                    <  1:315388‑M2/27‑1 (MQ=255)`  
                               `tggttgtaacgtccgttgtaattaatcctgaggcagactctaagggcttacagcgattcctagcgaaacgctttaagtaccttgcggacctccacgattgcgattgggtgtctcgttgtaagcatgaaggcgagacaatgcgtgtatactttaaggaggtataagttatgggtaagaaagttaagaaggccgtgaagaaagtcaccaagtccgttaagaaagtcgttaaggaaggggctcgtccggttaaacaggttgctggcggtctGGCTGGTGGTACTGGTGAAGCACAGATGGTGGa                                                                                                                                                                                                                                                                              <  1:22272‑M2/33‑1 (MQ=255)`  
                                `ggatgtaacgtccgttgtcattaatcatgaggcagactctaagggcttacagcgattcctagcgaaacgctttaagtaccttgcggaactccacgattgcgattgggtgtctcgttgtaagcatgaaggcgacacaatgcgtgtatactttaaggaggtataagttatgggtaagaaagttaagaaggccgtgaagaaagtcaccaagtccgttaagaaagtcgttaaggaaggggctcgtccggttaaacaggttgctggcggtctGGCTGGTGGTACTGGTGAAGCACAGATGGTGGaa                                                                                                                                                                                                                                                                             >  1:177820‑M2/268‑301 (MQ=255)`  
                                  `tgtgtataagagacagcattaatcctgaggcagactctaagggcttacagcgattcctagcgaaacgctttaagtaccttgcggaactccacgattgcgattgggtgtctcgttgtaagcatgaaggcgagacaatgcgtgtatactttaaggaggtataagttatgggtaagaaagttaagaaggccgtgaagaaagtcaccaagtccgttaagaaagtcgttaaggaaggggctcgtccggttaaacaggttgctggcggtctGGCTGGTGGTACTGGTGAAGCACAGATGGTGGAAGt                                                                                                                                                                                                                                                                           <  1:126072‑M2/36‑1 (MQ=255)`  
                                       `gTCATTAATCCTGAGGCAGACTCTAAGGGCTTACAGCGATTCCTAGCGAAACGCTTTAAGTACCTTGCGGAACTCCACGATTGCGATTGGGTGTCTAGTTGTAAGCATGAAGGCGAGACAATGCGTGTATACTTTAAGGAGGTATAAGTTATGGGTAAGAAAGTTAAGAAGGCCGTGAAGAAAGTCACCAAGTCCGTTAAGAAAGTCGTTAAGGAAGGGGCTCGTCCGGTTAAACAGGTTTCTGGCGGTCTAGCTGGTCTGGCTGGTGGTACTGGTGAAGCACAGATGGTGGAAGTACcac                                                                                                                                                                                                                                                                      >  1:134759/1‑301 (MQ=255)`  
                                       `gTCATTAATCCTGAGGCAGACTCTAAGGGCTTACAGCGATTCCTAGCGAAACGCTTTAAGTACATTGCGGAACTCCACGATTGCGATTGGGTGTCTCGTTGTAAGCATGAAGGCGAGACAATGCGTGTATACTTTAAGGAGGTATAAGTTATGGGTAAGAAAGTTAAGAAGGCCGTGAAGAAAGTCACCAAGTCCGTTAAGAAAGTCGTTAAGGAAGGGGCTCGTCCGGTTAAACAGGTTGCTGGCGGTCTAGCTGGTCTGGCTGGTGGTACTGGTGAAGCACAGATGGTGGAAGTACcac                                                                                                                                                                                                                                                                      <  1:227225/301‑1 (MQ=255)`  
                                       `acgtccgttgtcattaatcctgaggcagactctaagggcttacagcgattcctagcgaaacgctttaagtaccttgcggaactgcacgattgcgattgggtgtctcgttgtaagcatgaaggcgagacaatgcgtgtatactttaaggaggtataagttatgggtaagaaagttaagaaggccgtgaagaaagtcaccaagtccgttaagaaagtcgttaaggaaggggctcgtccggttaaacaggttgctggcggtctGGCTGGTGGTACTGGTGAAGCACAGATGGTGGAAGTACcac                                                                                                                                                                                                                                                                      <  1:66910‑M2/41‑1 (MQ=255)`  
                                        `cgtccgttgtcattaatcctgaggcagactctaagggcttacagcgattcctagcgaaacgctttaagtaccttgcggaactccacgattgcgattgggtgtctcgttgtaagcatgaaggcgagacaatgcgtgtatactttaaggaggtataagttatgggtaagaaagttaagaaggccgtgaagaaagtcaccaagtccgttaagaaagtagttaaggaaggggctcgtccggttaaacaggttgctggcggtctGGCTGGTGGTACTGGtgaagcacagatgctgtctcttataca                                                                                                                                                                                                                                                                     >  1:55116‑M2/260‑290 (MQ=255)`  
                                         `aaTTAATACTGAGGCAGACTCTAAGGGCTTACAGCGATTCCTAGCGAAACGCTTTAAGTAACTTGCGGAACTCCACGATTGCGATTGGGTGTCTCGTTGTAAGCATGAAGGCGAGACAATGCGTGTATACTTTAAGGAGGTATAAGTTATGGGTAAGAAAGTTAAGAAGGCCGTGAAGAAAGTCACCAAGTCCGTTAAGAAAGTCGTTAAGGAAGGGGCTCGTCCGGTTAAACAGGTTTCTGGCGGTCTAGCTGGTCTGGCTGGTGGTACTGGTGAAGCACAGATGGTGGAAGTACCACaa                                                                                                                                                                                                                                                                    <  1:69487/300‑1 (MQ=255)`  
                                               `tCCTGAGGCAGACTCTAAGGGCTTACAGCGATTCCTAGCGAAACGCTTTAAGTACCTTGCGGAACTCCACGATTGCGATTGGGTGTCTCGTTGTAAGCATGAAGGCGAGACAATGCGTGTATACTTTAAGGAGGTATAAGTTATGGGTAAGAAAGTTAAGAAGGACGTGAAGAAAGTCACCAAGTCCGTTAAGAAAGTCGTTAAGGAAGGGGCTAGTCCGGTTAAACAGGTTGCTGGCGGTCTAGCTGGTCTGGCT‑GTGGTACTGGTGAAGCACAGATGGTGGAAGTACGAAAAGCGGCCg                                                                                                                                                                                                                                                             >  1:299847/1‑301 (MQ=255)`  
                                               `tCCTGAGGCAGACTCTAAGGGCTTACAGCGATTCCTAGCGAAACGCTTTAAGTACCTTGCGGAACTCCACGATCGCGATTGGGTGTCTCGTTGTAAGCATGAAGGCGAGACAATGCGTGTATACTTTAAGGAGGTATAAGTTATGGGTAAGAAAGTTAAGAAGGCCGTGAAGAAAGTCACCAAGTACGTTAAGAAAGTCGTTAAGGAAGGGGCTCGTCCAGTTAAACAGGTTTCTGGCGGTCTAGCTGGTCTGGCTGGTGGTACTGGTGAAGCACAGATGGTGGAAGTACCACAAGCTGcc                                                                                                                                                                                                                                                              >  1:14047/1‑301 (MQ=255)`  
                                                  `cattaatcctgaggcagactctaagggcttacagcgattcctagcgaaacgctttaagtaccttgcggaactccacgattgcgatttggtgtctcgttgtaagcatgaaggcgagactctgcgtgtatactttaaggaggtatcagttctgggtaagaaagttaagaaggccgtgaagcaagccaccaattccgttaagaaagtcgttaaggaaggggcttgtccggttaaacaggttgctggcgttttTGCTGGTGGTACTGGTGAACCTCAGATGGTGGACGTACCACaagctgactct                                                                                                                                                                                                                                                           >  1:171991‑M2/250‑296 (MQ=255)`  
                                                   `attaatcctgaggcagactctaagggcttacagcgattcctagcgaaacgctttaagtaccttgcggaactccacgattgcgattgggtgtctcgttgtaagcatgaaggcgagacaatgcgtgtatactttaaggaggtataagttatgggtaagaaagttaagaaggccgtgaagaaagtcaccaagtccgttaagaaagtcgttaaggaaggggctcgtccggttaaacaggttgctggcggtctGGCTGGTGGTACTGGTGAAGCACAGATGGTGGAAGTACCACAAGCTGCCGcac                                                                                                                                                                                                                                                          <  1:283754‑M2/53‑1 (MQ=255)`  
                                                     `taatcctgaggcagactctaagggcttacagcgattcctagcgaaacgctttaagtaccttgcggaactccacgattgcgattgggtgtctcgttgtaagcatgaaggcgagacaatgcgtgtatactttaaggaggtataagttatgggtaagaaagttaagaaggccgtgaagaaagtcaccaagtccgttaagaaagtcgttaaggaaggggctcgtccggttaaacaggttgctggcggtctGGCTGGTGGTACTGGTGAAGCACAGATGGTGGAAGTACCACAAGCTGCCGCACAg                                                                                                                                                                                                                                                        <  1:27161‑M2/55‑1 (MQ=255)`  
                                                       `aggACTCTAAGGGCTTACAGCGATTCCTAGCGAAACGCTTTAAGTACCTTGCGGAACTCCACGATTGCGATTGGGTGTCTCGTTGTAAGCATGAAGGCGAGACAATGCGTGTATACTTTAAGGAGGTATAAGTTATGGGTAAGAAAGTTAAGAAGGCCGTGAAGAAAGTCACCAAGTCCGTTAAGAAAGTCGTTAAGGAAGGGGCTCGTCCGGTTAAACAGGTTTCTGGCGGTCTAGCTGGTCTGGCTGGTGGTACTGGTGAAGCACAGATGGTGGAAGTACCACAAGCTGCCGCACAGAt                                                                                                                                                                                                                                                      <  1:47951/299‑1 (MQ=255)`  
                                                             `aggcaggttttaagggcttacagcggttcctagcgaaacgctttaagtaccttgcggaactccacgattgcgattgggtgtctcgttgtaagcatgaaggcgagacaatgcgtgtatactttaaggaggtataagttatgggtaagaaagttaagaaggccgtgaagaaagtcaccaagtccgttaagaaagtcgttaaggaaggggctcgtccggttaaacaggttgctggcggtctGGCTGGTGGTACTGGTGAAGCACAGATGGTGGAAGTACCACAAGCTGCCGCACAGATTGTTGa                                                                                                                                                                                                                                                <  1:119021‑M2/63‑1 (MQ=255)`  
                                                              `ggcagactctaagagcttacggcgattcctagcgaaacgctttaagtaccttgcagaactccacgattgcgattgggtgtctcgttgtaagcatgaaggctagacaatgcgtgtatactttaaggaggtataagttatgggtaagaaagttaagaaggccgtgaagaaagtcaccaagtccgttaagaaagtcgttaaggaaggggctcgtccggttaaacaggttgctggcggtctGGCTGGTGGTACTGGTGAAGCACAGATGGTGGAAGTACCACAAGCTGCCGCACAGATTGTTGAc                                                                                                                                                                                                                                               <  1:191015‑M2/64‑1 (MQ=255)`  
                                                                    `ctctaagggcttacatcgattcctagcgaaacgctttaagtaccttgcggaactccacgattgcgattgggtgtctcgttgtaagcatgaaggcgagacaatgcgtgtatactttaagtaggtataagttatgggtaagaaagttaagaaggccgtgaagaaagtcaccaagtccgttaagaaagtcgttaaggaaggggctcgtccggttaaacaggttgctggcggtctGGCTGGTGGTACTGGTGAAGCACAGATGGTGGAAGTACCACAAGCTGCCGCACAGATTGttgacgctgtc                                                                                                                                                                                                                                         >  1:93777‑M2/232‑296 (MQ=255)`  
                                                                           `ggcttacagcgattcctagcgaaacgctttaagtaccttgcggaactccacgattgcgattgggtgtctcgttgtaagcatgaaggcgagacaatgcgtgtatactttaaggaggtataagttatgggtaagaaagttaagaaggccgtgaagaaagtcaccaagtccgttaagaaagtcgttaaggaaggggctcgtccggttaaacaggttgctggcggtctGGCTGGTGGTACTGGTGAAGCACAGATGATGGAAGTACCACATGATGCCGCACAGATTGTTGACGTACCTGGGAAag                                                                                                                                                                                                                                  >  1:86817‑M2/225‑301 (MQ=255)`  
                                                                                     `gattcctagcgaaacgctttaagtaccttgcggaactccacgattgcgattgggtgtctcgttgtaagcatgaaggcgagacaatgcgtgtatactttaaggaggtataagttattggtaagaaagttaagaaggccgtgaagaaagtcaccaagtccgttaagaaagtcgttaaggaaggggctcgtccggttaaacaggttgctggcggtctGGCTGGTGGTACTGGTGAAGCACAGATGGTGGAAGTACCACAAGCTGCCGCACAGATTGTTGACGTACCTGAGAAAGAGGTTTCCAc                                                                                                                                                                                                                        <  1:11611‑M2/87‑1 (MQ=255)`  
                                                                                     `gattcctagagaaacgctttaagtaccttgcggaactccacgattgcgattgggtgtctcgttgtaagcatgaagacgagacaatgcgtgtatactttaaggaggtataagttatgggtaagaaagttaagaaggccgtgaagaaagtcaccaagtccgttaagaaagtcgttaaggaaggggctcgtccggttaaacaggttgctggcggtctGGCTGGTGGTACTGGTGAAGCACAGATGGTGGAAGTACCACAAGCTGCCGCACAGATTGTTGACGTACCTGAGAAAGAGGTTTCCAc                                                                                                                                                                                                                        <  1:89284‑M2/87‑1 (MQ=255)`  
                                                                                     `gagacagCTTTAAGTACCTTGCGGAACTCCACGATTGCGATTGGGGGTCTCGTTGGAAGCATGAAGGCGAGACAATGCGTGTATACTTTAAGGATGTATAAGTTATGGGTAAGAAAGTGAAGAAGGCCGTGAAGAAAGTCACCAAGTCCGTTAAGAAAGTCGTTAAGGAAGGGGCTCGTCCGGTTAAACAGGTTTCTGGCGGTCTAGCTGGTCTGGCTGGTGGTACTGGTGAAGCACAGATGGTGGAAGTACCACAAGCTGCCGCACAGATTGTTGACGTACCTGAGAAAGAGGTTTCCAc                                                                                                                                                                                                                        <  1:25756/295‑1 (MQ=255)`  
                                                                                               `ggtataagagacagaccttgcggaactccacgattgcgattgggtgtctcgttgtaagcatgaaggctagacaatgcgtgtatactttaaggaggtataagttatgggtaagaaagttaagaaggccgtgaagaaagtcaccaagtccgttaagaaagtcgttaaggaaggggctcgtccggttaaacaggttgctggcggtctGGCTGGTGGTACTGGTGAAGCACAGATGGTGGAAGTACCACAAGCTGCCGCACAGATTGTTGACGTACCTGAGAAAGAGGTTTCCACTGAGGACGaa                                                                                                                                                                                                              <  1:274800‑M2/97‑1 (MQ=255)`  
                                                                                                `aaacgctttaagtaactggcggaactccacgattgcgattgggtgtctcgttgtaagcatgaaggcgagacaatgcgtgtatactttaaggaggtataagttatgggtaagaaagttaagaaggccgtgaagaaagtcaccaagtccgttaagaaagtcgttaaggaaggggctcgtccggttaaacaggttgctggcggtctAGCTGGTGGTACTGGTGAAGCACAGATGGTGGAAGTACCACAAGCTGCCGCACAGATTGTTGACGTACCTGAGAAAGAGGTTTccactgagctgtctc                                                                                                                                                                                                             <  1:167126‑M2/98‑8 (MQ=255)`  
                                                                                                  `acgctttaagtaccttgcggaactccacgattgcgattgggtgtctcgttgtaagcatgaaggcgagacaatgcgtgtatactttaaggaggtataagttatgggtaagaaagttaagaaggccgtgaagaaagtcaccaagtccgttaagaaagtcgttaaggaaggggctcgtccggttaaacaggttgctggcggtctGGCTGGTGGTACTGGTGAAGCACAGATGGTGGAAGTACCACAAGCTGCCGCACAGATTGTTGACGTACCTGAGAAAGAGGTTTCCACTGAGGACGAAGca                                                                                                                                                                                                           >  1:266942‑M2/202‑301 (MQ=255)`  
                                                                                                   `cgctttaagtaccttgcggaactccacgattgcgattgggtgtctcgttgtaagcatgaaggcgagacaatgcgtgtatactttaaggaggtataagttatgggtaagaaagttaagaaggccgtgaagaaagtcaccaagtccgttaagaaagtcgttaaggaaggggctcgtccggttaaacaggttgctggcggtctGGCTGGTGGTACTGGTGAAGCACAGATGGTGGAAGTACCACCAGCTGCCGCACAGATTGTTGACGTACCTGATAAAGAGGTTTCCACTGAGGACGAAGcac                                                                                                                                                                                                          >  1:64753‑M2/201‑301 (MQ=255)`  
                                                                                                         `aagtaccttgcggaactccacgattgcggttgggtgtctcgttgtaagcatgaaggcgagacaatgcgtgtatactttaaggaggtataagttatgggtaagaaagttaagaaggccgtgaagaaagtcaccaagtccgttaagaaagtcgttaaggaaggggctcgtccggttaaacaggttgctggcggtctGGCTGGTGGTACTGGTGAAGCACAGATGGTGGAAGTACCACAAGCTGCCGCACAGATTGTTGACGTACATGAGAAAGAGGTTTCCACTGAGGACGAAGCacagacag                                                                                                                                                                                                    <  1:240019‑M2/107‑1 (MQ=255)`  
                                                                                                           `ataccttgcggaactccacgattgcggttgggtgtctcgttgtaagcatgaaggggagacaatgcgtgtatactttaaggaggtataagttatgggtaagaaagttaagaaggccgtgaagaaagtcaccaagtccgttaagaaagtcgttaaggaaggggctcgtccggtaaaacaggttgctggcggtctGGCTGGTGGTACTGGTGAAGCACAGATGGTGGAAGTACCACAAGCTGCCGCACAGATTGTTGACGTACCTGAGAAAGAGGTTTCCACTGAGGACGAAGCACAGACAGaa                                                                                                                                                                                                  <  1:105713‑M2/109‑1 (MQ=255)`  
                                                                                                             `accttgcggaactccacgattgcgattgggtgtctcgttgtaagcatgaaggcgagacaatgcgtgtatactttaaggaggtataagttatgggtaagaaagttaagaaggccgtgaagaaagtcaccaagtccgttaagaaagtcgttaaggaaggggctcgtccggttaaacaggttgctggcggtctGGCTGGTGGTACTGGTGAAGCACAGATGGTGGAAGTACCACAAGCTGCCGCACAGATTGTTGACGTACCTGAGAAAGAGGTTTCCACTGAGGACGAAGCACAGACAGAAAg                                                                                                                                                                                                <  1:158402‑M2/111‑1 (MQ=255)`  
                                                                                                                             `cgattgcgattgggtgtctcgttgtaagaatgaaggcgagacaatgcgtgtatactttaaggaggtataagttatgggtaagaaagttaagaaggccgtgaagaaaggcaccaagtccgttaagaaagtcgttaaggaaggggctcgtcaggttaaacaggttgctggcggtctGGCTGGTGGTACTGGTGAAGCACAGATGGTGGAAGTACCACAAGCTGCCGCACAGATTGTTGACGTACCTGAGAAAGAGGTTTCCACTGAGGACGAAGCACAGACAGAAAGCGGACGCAAGAAAGCt                                                                                                                                                                                <  1:110286‑M2/127‑1 (MQ=255)`  
                                                                                                                               `attgcgattgggtgtctcgttgtaagcatgaaggcgagacaatgcgtgtatactttaaggaggtataagttatgggtaagaaagttaagaaggccgtgaagaaagtcaccaagtccgttaagaaagtcgttaaggaaggggctcgtccggttaaacaggttgctggcggtctGGCTGGTGGTACTGGTGAAGCACAGATGGTGGAAGTACCACAAGCTGCCGCACAGATTGTTGACGTACCTGAGAAAGAGGTTTCCACTGATGACGAAGCACAGACAGAAAGCGGACGCAAGAAAGCTCg                                                                                                                                                                              >  1:236376‑M2/173‑301 (MQ=255)`  
                                                                                                                                   `cgattgggtgtctcgttgtaagcatgaaggcgagacaatgcgtgtatactttaaggaggtataagttatgggtaagaaagttaagaaggccgtgaagaaagtcaccaagtccgttaagaaagtcgttaaggaaggggctcgtccggttaaacaggttgctggcggtctGGCTGGTGGTACTGGTGAAGCACAGATGGTGGAAGTACCACAAGCTGCCGCACAGATTGTTGACGTACCTGTGCAAGAGGTTTCCACTGAGGACGAAGCACAGACAGCAAGCGGACGCAAGCACGCTCGTGCt                                                                                                                                                                          >  1:262775‑M2/169‑301 (MQ=255)`  
                                                                                                                                            `gtctcgttgtaagcatgaaggcgagacaatgcgtgtatactttaaggaggtataagttatgggtaagaaagttaagaaggacgtgaagaaagtcaccaagtccgttaagaaagtcgttaaggaaggggctcgtccggttaaacaggttgctggcggtctGGCTGGTGGTACTGGTGAAGCACAGATGGTGGCAGTACCACAAGCTGCCGCACAGATTGTTGACGTACCTGAGAAAGAGGTTTCCACTGAGGACGAAGCACAGACAGAAAGCGGACGCAAGAAAGCTCGTGCTGGCGGTAAg                                                                                                                                                                 >  1:248694‑M2/160‑301 (MQ=255)`  
                                                                                                                                             `tctcgttgtaagcatgaaggcgagacaatgcgtgtatactttaaggaggtataagttatgggtaagaaagttaagaaggccgtgaagaaagtcaccaagtccgttaagaaagtcgttaaggaaggggctcgtccggttaaacaggttgctggcggtctGGCTGGTGGTACTGGTGAAGCACAGATGGTGGAAGTACCACAAGCTGCCGCACAGATTGTTGACGTACCTGAGAAAGAGGTTTCCACTGAGGACGAAGCACAGACAGAAAGCGGACGCAAGAAAGCTCGTGCTGGCGGTAAGa                                                                                                                                                                >  1:79810‑M2/159‑301 (MQ=255)`  
                                                                                                                                                     `aGGCGAGACAATGCGTGTATACTTTAAGGAGGTATAAGTTATGGGTAAGAAAGTTAAGAAGGCCGTGAAGAAAGTCACCAAGTACGTTAAGAAAGTCGTTAAGGAAGGGGCTCGTCCGGTTAAACAGGTTTCTGGCGGTCTAGCTGGTCTGGCTGGTGGTACTGGTGAAGCACAGATGGTGGAAGTACCACAAGCTGCCGCACAGATTGTTGACGTACCTGAGAAAGAGGTTACCACTGAGGACGAAGCACAGACAGAAAGCGGACGCAAGAAAGCTCGTGCTGGCGGTAAGAAATCCTTg                                                                                                                                                        >  1:107342/1‑301 (MQ=255)`  
                                                                                                                                                         `gagaCAATGCGTGTATACTTTAAGGAGGTATAAGTTATGGGTAAGAAAGTTAAGAAGGCCGTGAAGAAAGTCACCAAGTCCGTTAAGAAAGTCGTTAAGGAAGGGGCTCGTCCGGTTAAACAGGTTTCTGGCGGTCTAGCTGGTCTGGCTGGTGGTACTGGTGAAGCACAGATGGTGGAAGTACCACAAGCTGCCGCACAGATTGTTGACGTACCTGAGAAAGAGGTTTCCACTGAGGACGAAGCACAGACAGAAAGCGGACGCAAGAAAGCTCGTGCTGGCGGTAAGAAATCCTTGAgtg                                                                                                                                                    <  1:329680/301‑1 (MQ=255)`  
                                                                                                                                                              `aggcgggacaatgcaggtatactttaaggagttataagttatgggtaagaaagttaagaaggccgtgaagaaagtcaccaagtccgttaagaaagtcgttaaggaaggggctcgtccggttaaacaggttgctggcggtctGGCTGGTGGTACTGGTGAAGCACAGATGGTGGAAGTACCACAAGCTGCCGCACAGATTGTTGACGTACCTGAGAAAGAGGTTTCCACTGAGGACGAAGCACAGACAGAAAGCGGACGCAAGAAAGCTCGTGCTGGCGGTAAGAAATCCTTGAGTGTAGcc                                                                                                                                               <  1:320105‑M2/160‑1 (MQ=255)`  
                                                                                                                                                                 `gCGTGTATACTTTAAGGAGGTATAAGTTATGGGTAAGAAAGTTAAGAAGGCCGTGAAGAAAGTCACCAAGTCCGTTAAGAAAGTCGTTAAGGAAGGGGCTCGTCCGGTTAAACAGGTTGCTGGCGGTCTAGCTGGTCTGGCTGGTGGTACTGGTGAAGCACAGATGGTGGAAGTACCACAAGCTGCCGCACAGATTGTTGACGTACCTGAGAAAGAGGTTTCCACTGAGGACGAAGCACAGACAGAAAGCGGACGCAAGAAAGCTCGTTCTGGCGGTAAGAAATCCTTGAGTGTAGCCCGt                                                                                                                                            >  1:61967/1‑301 (MQ=255)`  
                                                                                                                                                                      `aaatgcgtgtatactttaaggaggtataagttatgggtaagaaagttaagaaggccatgaagaaagtcaacaagtccgttaagaaagtcgttaaggaaggggctcgtccggttaaacaggttgctggcggtctGGCTGGTGGTACTGGTGAAGCACAGATGGTGGAAGTACCACAAGCTGCCGCACAGATTGTTGACGTACCTGAGAAAGAGGTTTCCACTGAGGACGAAGCACAGACAGAAAGCGGACGCAAGAAAGCTCGTGCTGGCGGTAAGAAATCCTTGAGTGTAGCCCGTAGCTc                                                                                                                                       <  1:434‑M2/168‑1 (MQ=255)`  
                                                                                                                                                                          `gcgtgtatactttaaggaggtataagttatgggtaagaaagttaagaaggccgtgaagaaagtcaccaagtccgttaagaaagtcgttaaggaagtggctcgtccggttaaacaggttgctggcggtctGGCTGGTGGTACTGGTGAAGCCCAGATGGTGGAAGTACCACAAGCTGCCGCACAGATTGTTGACGTACCTGAGAACGAGGTTTCCCCTGAGGGCGCAGCACAGACAGAAAGCGGACGCAAGAAAGCTCGTGCTGGCGGTAAGCAAACCTTGAGTGTAGCCTGTAGCTCCGGt                                                                                                                                   >  1:27856‑M2/130‑301 (MQ=255)`  
                                                                                                                                                                          `cTTTAAGGAGGTATAAGTTATGGGTAAGAAAGTTAAGAAGGCCGTGAAGAAAGTCACCAAGTCCGTTAAGAAAGTCGTTAAGGAAGGGGCTCGTCCGGTTAAACAGGCTGCTGGCGGTCTAGCTGGTCTGGCTGGTGGTACTGGTGAAGCACATATGGTGGAAGTACCACAAGCTGCCGCACAGATTGTTGACGTACCTGAGAAAGAGGTTTCCACTGAGGACGAAGCACAGACAGAAAGCGGACGCAAGAAAGCTCGTGCTGGCGGTAAGAAATCCTTGAGTGTAGCCCGTAGcctgtcc                                                                                                                                   >  1:40001/1‑295 (MQ=255)`  
                                                                                                                                                                           `ttgaaggggggttaAGTTATGGGAAAAAAAGGTAGGAAGGCCGTGAAGAAAGGAAACAAGTCCGTTAAGAAAGGCGTTAAGGAATGGGATCGTCCGGTTAAACAGGTTTCTGGCGGTCTAGCTGGTCTGGCTGGTGGTACTGGTGAAGCAAAGATGGTGGAAGTACCACAAGCTGCCGCACAGATTGTTGACGTACCTGAGAAAGAGGTTTCCACTGAGGACGAAGCACAGACAGAAAGCGGACGCAAGAAAGCTCGTGCTGGCGGTAAGAAATCCTTGAGTGTAGCCCGTAGCTCCGGTg                                                                                                                                  <  1:162159/289‑1 (MQ=255)`  
                                                                                                                                                                           `cgtgtatactttaaggaggtataagttatgggtaagaaagttaataaggccgtgaagaaagtcaccaagtccgttaagaaagtcgttaaggaaggggctcgtccggttaaacaggttgctggcggtctGGCTGGTGGTACTGGTGAAGCACATATGGTGGAAGTACCACACGCTGCCGCACAGATTGTTGACGTACCTGAGAAAGAGGTTTCCACTGAGGACGAAGCACATACAGAAAGCGGACGCAAGAAAGCTCGTGATGTCGGTAAGAAATCCTTGAGTGTAGCCCGTAGCTCCGGTg                                                                                                                                  >  1:74058‑M2/129‑301 (MQ=255)`  
                                                                                                                                                                           `cgtgtatactttaaggaggtataagttatgggtaagaaagttaagaaggccgtgaagaaagtcaccaagtccgttaagaaagtcgttaaggaaggggctcgtccggttaaacaggttgctggcggtctGGCTGGTGGTACTGGTTAAGCACAGATGGTGGAAGTACCACAAGCTGCCGCACAGATTGTTGACGTACCTGAGAAAGAGGTTTCCACTGCGGACGAATCACATACAGAAAGCGGACGCACGAAAGCTCGTGCTGgcggtaagcaatccttgagtgctgtctcttatacacatc                                                                                                                                  >  1:128622‑M2/129‑283 (MQ=255)`  
                                                                                                                                                                            `gtgtatactttaaggaggtataagttaggggtaagaaagttaagaaggccgtgaagaaagtcaccaagtccgttaagaaagtcgttaaggaaggggctcggccggttaaacaggtggctggcggtctAGCTGGTGGTACTGGTGAAGCACAGATGGTGGAAGTACCACAAGCTGCCGAACAGATTGTTGACGTACCTGAGAAAGAGGTTTCCACTGAGGACGAAGCACAGACAGAAAGCGGACGCAAGAAAGCTCGTGCTGGCGGTAAGAAATCCTTGAGTGTAGCCCGTAGCTCCGGTgg                                                                                                                                 <  1:246161‑M2/174‑1 (MQ=255)`  
                                                                                                                                                                                   `ctttaaggaggtataagttatgggtaagaaagttaagaaggccgtgaagaaagtcaccaagtccgttaagaaagtcgttaaggaaggggctcgtccggttaaacaggttgctggcggtctGGCTGGTGGTACTGGTGAAGCACAGATGGTGGAAGTACCACAAGCTGCCGCACAGATTGTTGACGTACCTGAGAAAGAGGTTTCCACTGAGGACGAAGCACAGACAGAAAGCGGACGCAAGAAAGCTCGTGCTGGCGGtaagaaatccttgagtgtagccctgtctcttatacacatctcc                                                                                                                          >  1:112341‑M2/121‑281 (MQ=255)`  
                                                                                                                                                                                    `gTATAAGTTATGGGTAAGAAAGTTAAGAAGGCCGTGAAGAAAGTCACCAAGTCCGTTAAGAAAGTCGTTAAGGAAGGGGCTCGTCCGGTTAAACAGGTTTCTGGCGGTCTAGCTGGTCTGGCTGGTGGTACTGGTGAAGCACAGATGGTGGAAGTACCACAAGCTGCCGCACAGATTGTTGACGTACCTGAGAAAGAGGTTTCCACTGAGGACGAAGCACAGACAGAAAGCGGACGCAAGAAAGCTCGTGCTGGCGGTAAGAAATCCTTGAGTGTAGCCCGTAGCTCCGGTGGCGGTATCa                                                                                                                         >  1:291464/1‑301 (MQ=255)`  
                                                                                                                                                                                         `aGTTATTGGTAAGAAAGTTAAGAAGGCCGTGAAGAAAGTCACCAAGTCCGTTAAGAAAGTCGTTAAGGAAGGGGCTCGTCCGGTTAAACAGGTTTCTGGCGGTCTAGCTGGTCTGGCTGGTGGTACTGGTGAAGCACAGATGGTGGAAGTACCACAAGCTGCCGCACAGATTGTTGACGTACCTGAGAAAGAGGTTTCCACTGAGGACGAAGCACAGACAGAAAGCGGACGCAAGAAAGCTCGTGCTGGCGGTAAGAAATCCTTGAGTGTAGCCCGTAGCTCCGGTGGCGGTATCAACAtt                                                                                                                    >  1:229868/1‑301 (MQ=255)`  
                                                                                                                                                                                            `ggtataagttatgggtaagaaagttaagaaggccgtgaagaaagtcaccaagtccgttaagaaagtcgttaaggaaggggctcgtccggttaaacaggttgctggcggtctGGCTGGTGGTACTGGTGAAGCACAGATGGTGGAAGTACCACAAGCTGCCGCACAGATTGTTGACGTACCTGAGAAAGAGGTTTCCACTGAGGACGAAGCACAGACAGAAAGCGGACGCAAGAAAGCTCGTGCTGGCGGTAAGAAATCCTTGAGTGTAGCCCGTAGCTCCGGTGGCGGTATCAACATTTaa                                                                                                                 >  1:103732‑M2/112‑301 (MQ=255)`  
                                                                                                                                                                                            `ggtataagttatgggtaagaaagttaagaaggccgtgaagaaagtcaccaagtccgttaagaaagtcgttaaggaaggggctcgtccggttaaacaggttgctggcggtctGGCTGGTGGTACTGGTGAAGCACAGATGGTGGAAGTACCACAAGCTGCCGCACAGATTGTTGACGTACCTGAGAAAGAGGTTTCCACTGAGGACGAAGCACAGACAGAAAGCGGACGCAAGAAAGCTCGTGCTGGCGGTAAGAAATCCTTGAGTGTAGCCCGTAGCTCCGGTGGCGGTATCAACATTTaa                                                                                                                 >  1:169406‑M2/112‑301 (MQ=255)`  
                                                                                                                                                                                            `ggtataagttatgggtaagaaagttaagaaggccgtgaagaaagtcaccaagtccgttaagaaagtcgttaaggaaggggctcgtccggttaaacaggttgctggcggtctGGCTGGTGGTACTGGTGAAGCACAGATGGTGGAAGTACCACAAGCTGCCGCACAGATTGTTGACGTACCTGAGAAAGAGGTTTCCACTGAGGACGAAGCACAGACAGAAAGCGGACGCAAGAAAGCTCGTGCTGGCGGTAAGAAATCCTTGAGTGTAGCCCGTAGCTCCGGTGGCGGTATAAacattctg                                                                                                                 >  1:260509‑M2/112‑298 (MQ=255)`  
                                                                                                                                                                                              `tataagttatgggtaagaaagttaagaaggccgtgaagaaagtcaccaagtccgttaagaaagtcgttaaggaaggggctcgtccggttaaaaaggttgctggcggtctGGCTGGTGGTACTGGTGAAGCACAGATGGTGGAAGTACCACAAGCTGCCGCACAGATTGTTGACGTACCTGAGAAAGAGGTTTCCACTGAGGTCGAAGCACAGACAGAAAGCGGACGCAAGAAAGCTCGTTCTGGCGGTAAGCAATCCTTGAGTGTAGCCCGTCGCtccggtggctgtctcttatacaattt                                                                                                               >  1:295893‑M2/110‑290 (MQ=255)`  
                                                                                                                                                                                                      `atgggtaagaaagttaagaaggccgtgaagaaaggcaccaagtccgttaagaaaggcgttaaggaaggggctcgtccggttaaacaggttgctggcggtctGGCTGGTGGTACTGGTGAAGCACAGATGGTGGAAGTACCACAAGCTGCCGCACAGATTGTTGACGTACCTGAGAAAGAGGTTTCCACTGAGGACGAAGCACAGACAGAAAGCGGACGCAAGAAAGCTCGTGCTGGCGGTAAGAAATCCTTGAGTGTAGCCCGTAGCTCCGGTGGCGGTATTAACATTTAATCAGGAGGtt                                                                                                       <  1:233319‑M2/200‑1 (MQ=255)`  
                                                                                                                                                                                                        `gggtaagaaagttaagaaggccgtgaagaaagtcaccaagtccgttaagaaagtcgttaaggaaggggctcgtccggttaaacaggttgctggcggtctGGCTGGTGGTACTGGTGAAGCACAGATGGTGGAAGTACCACAAGCTGCCGCACAGATTGTTGACGTACCTGAGAAAGAGGTTTCCACTGAGGACGAAGCACAGACAGAAAGCGGACGCAAGAAAGCTCGTGCTGGCGGTAAGAAATCCTTGAGTGTAGCCCGTAGCTCCGGTGGCGGTATCAACATTTAATCAGGAGGTTAt                                                                                                     <  1:142706‑M2/202‑1 (MQ=255)`  
                                                                                                                                                                                                              `gaaagttaagaaggccgtgaagaaagtaaccaagtccgttaagaaagtcgttaaggaaggggctcgtccggttaaacaggttgctggcggcctGGCTGGTGGTACTGGTGAAGCACAGATGGTGGACGTACCACAAGCTGCCGCACAGATTGTTGACGTACCTGAGAAAGAGGTTTCCACTGAGGACGAAGCACAGACAGAAAGCGGACGCAAGAAAGCTCGTGCTGGCGGTAAGAAATCCTTGAGTGTAGCCCGTAGCTCCGGTGGCGGGGTCaacatgtaatcaggagcggtctcttat                                                                                               >  1:35848‑M2/94‑290 (MQ=255)`  
                                                                                                                                                                                                               `aaatgtaagaaggacgttaagaaagttaacaagaccgttaagaaagtaggtaaggaaggggcgcgtccggttaaacaggttggtggcggtctGGCTGGTGGGAATGGTGAAGGACAGATGGTGGAAGGACAACAAGCTGCCGCACAGAGTGTAGACGTACCTGAGAAAGAGGTTTCCACTGAGGACGAAGCACAGACAGAAAGCGGACGTAAGAAAGCGAGGGCTGGCGGTAAGAAATCCTTGAGTGGAGTCAGTAGCTCCGGTGGCGGTATCAATATTTAATCAGTAGGTTATCGTGGaa                                                                                              <  1:28777‑M2/209‑1 (MQ=255)`  
                                                                                                                                                                                                                `aaattaagaaggccgtgaagaaagtcaccaagtccgttaagaaagtcgttaaggaaggggctcgtccggttaaacaggttgctggcggtctGGCTGGTGGTACTGGTGAAGCACAGATGGTGGAAGTACCACAAGCTGCCGCACAGATTGTTGACGTACCTGAGAAAGAGGTTTCCACTGAGGACGAAGCACAGACAGAAAGCGGACGCAAGAAAGCTCGTGCTGGCGGTAAGAAATCCTTGAGTGTAGCCCGTAGCTCCGGTGGCGGTATCAACATTTAATCAGGAGGTTATCGTGGAAg                                                                                             <  1:151642‑M2/210‑1 (MQ=255)`  
                                                                                                                                                                                                                  `gttaagaaggccgtgaagaaagtcaccaagtccgttaagaaagtcgttaaggaaggggctcgtccggttaaacaggttgctggcggtctGGCTGGTGGTACTGGTGAAGCACAGATGGTGGAAGTACCACAAGCTGCCGCACAGATTGTTGACGTACCTGAGAAAGAGGTTTCCACTGAGGACGAAGCACAGACAGAAAGCGGACGCAAGAAAGCTCGTGCTGGCGGTAAGAAATCCTTGAGTGTAGCCCGTAGCTCCGGTGGCGGTATCAACATTTAATCAGGAGGTTATCGTGGAAGAc                                                                                           >  1:251861‑M2/90‑301 (MQ=255)`  
                                                                                                                                                                                                                  `gttaagaaggccgtgaagaaagtcaccaagtccgttaagaaagtcgttaaggaaggggctcgtccggttaaacaggttgatggcggtctGGCTTGTGGTACTGGTGAAGCACAGATGGTGGAAGTACCACAAGCTGCCGCACAGATTGTTGACGTACCTGAGAAAGAGGTTTCCACTGAGGACGAAGCACAGACAGAAAGCGGACGCAAGAAAGCTCGTGCTGGCGGTAAGAAATCCTTGAGTGTAGCCCGTAGCTCCGGTGGCGGTATCAACATTTAATCAGGAGGTTATCGTGGAAGAc                                                                                           >  1:310150‑M2/90‑301 (MQ=255)`  
                                                                                                                                                                                                                     `aagaaggccgtgaagaaagtcaccaagtccgttaagaaagtcgttaaggaaggggctcgtccggttaaacaggttgctggcggtctGGCTGGTGGTACTGGTGAAGCACAGATGGTGGAAGTACAACAAGCTGCCGCACAGATTGTTGACGTACCTGAGAAAGAGGTTTCCACTGAGGACGAAGCACAGACAGAAAGCGGACGCAAGAAAGCTCGTGCTGGCGGTAAGAAATCCTTGAGTGTAGCCCGTAGCTCCGGTGGCGGTATCAACATTTAATCAGGAGGTTATCGTGGAAGACTGc                                                                                        <  1:96397‑M2/215‑1 (MQ=255)`  
                                                                                                                                                                                                                         `aggccgtgaagaaagtcaccaagtccgttaagaaagtcgttaaggaaggggctcgtccggttaaacaggttgctggcggtctGGCTGGTGGTACTGGTGAAGCACAGATGGTGGAAGTACCACAAGCTGCCGCACAGATTGTTGACGTACCTGAGAAAGAGGTTTCCACTGAGGACGAAGCACAGACAGAAAGCGGACGCAAGAAAGCTCGTGCTGGCGGTAAGAAATCCTTGAGTGTAGCCCGTAGCTCCGGTGGCGGTATCAACATTTAATCAGGAGGTTATCGTGGAAGACTGCATTg                                                                                    <  1:225360‑M2/219‑1 (MQ=255)`  
                                                                                                                                                                                                                           `gccgtgaagaaagtcaccaagtccgttaagaaagtcgttaaggaaggggctcgtccggttaaacaggttgctggcggtctGGCTGGTGGTACTGGTGAAGCACAGATGGTGGAAGTACCACAAGCTGCCGCACAGATTGTTGACGTACCTGATAAAGAGGTTTCCACTGAGGACGAAGCACAGACAGAAAGCGGACGCAAGAAAGCTCGTGCTGGCGGTAAGAAATCCTTGAGTGTAGCCCGTAGCTCCGGTGGCGGTATCAACATTTAATCAGGTGGTTATCGTTGAAGACTGCATTgat                                                                                  >  1:122539‑M2/81‑300 (MQ=255)`  
                                                                                                                                                                                                                            `aaGTCACCAGGGCCGGGAAGAAAGTCGTTAAGGAAGGGGCTCGGACGGGTAAACAGGTTGCTGGCGGTGTAGCTGGTCGGGCGGGTGGTACTGGTGAAGCACAGATGGTGGAAGTACCAAAAGCTGCCGCACAGATTGTTGACGTACCTGAGAAAGAGGTTTCCACTGATGACGAAGCACAGACAGAAAGCGGACGCAAGAAAGCTCGTGCTGGCGGTAAGAAATCCTTGAGTGTAGCCCGTAGCTCCGGTGGCGGTATCAACATTGAATCAGGAGGTGATCGTGGAAGACTGCATTGAAt                                                                                 <  1:77062/301‑1 (MQ=255)`  
                                                                                                                                                                                                                              `gTCACCAAGGCCGTTAAGAAAGTCGTTAAGGAAGGGGCTCGTTCGGGTAAACAGGTGTCTGGCGGTCTAGCTGGTCTGGCTGGTGGTACTGGTGAAGCACAGATGGTGGAAGTACCACAAGCTGCCGCACAGATTGTTGACGTACCTGAGAAAGAGGTTTCCACTGAGGACGAAGCACATACAGAAAGCGGACGCAAGAAAGCTCGTGCTGGCGGTAAGAAATCCTTGAGTGTAGCCCGTAGCTCCGGTGGCGGTATCAACATTTAATCAGGAGGTTATCGTGGAAGACTGCATTGAATgg                                                                               <  1:207076/301‑1 (MQ=255)`  
                                                                                                                                                                                                                                `cACCAAGTCCGTTAAGAAAGTCGTTAAGGAAGGGGCTCGTCCGGTTAAACAGGTTGCTGGCGGTCTAGCTGGTCTGGCTGGTGGTACTGGTGAAGCACAGATGGTGGAAGTACCACAAGCTGCCGCACAGATTGTTGACGTACCTGAGAAAGAGGTTTCCACTGAGGACGAAGCACAGACAGAAAGCGGACGCAAGAAAGCTCGTGCTGGCTGTAAGAAATCCTTGAGTGTAGCCCGTAGCTCCGGTGGCGGTATCAACATTTAATCAGGAGGTTATCGTGGAAGACTGCATTGAATGTAc                                                                             >  1:148868/1‑301 (MQ=255)`  
                                                                                                                                                                                                                                     `aGTCCGTTCAGAAAGTCGTTAAGGAGGGGGCGCGTCCGGTTAAACATGTTTCTGGCGGTCTAGCTGGTCTGGCTGGTGGTACTGGTGAAGCACAGATGGTGGAAGTACCACAAGCTGCCGCACAGATTGTTGACGTACCTGAGAAAGAGGTTTCCACTTAGGACGAAGCACAGACAGAAAGCGGACGCAAGAAAGCTCGTGCTGGCGGTAAGAAATCCTTGAGTGTAGCCCGTAGCTCCGGTGGCGGTATCAACATTTAATCAGGAGGTTATCGTGGAAGACTGCATTGAATGGACCGGAg                                                                        <  1:187915/301‑1 (MQ=255)`  
                                                                                                                                                                                                                                      `gTCCGTTAAGAAAGTCGTTAAGGAAGGTGCTCGTCCGGTTAAACAGGTTTCTGGCGGTCTAGCTGGTCTGGCTGGTGGTACTGGTGAAGCACAGATGGTGGAAGTACCACAAGCTGCCGCACAGATTGTTGACGTACCTGAGAAAGAGGTTTCCACTGATGACGAAGCACAGACAGAAAGCGGACGCAAGAAAGCTCGTGCTGGCGGTAAGAAATCCTTGAGTGTAGCCCGTAGCTCCGGTGGCGGTATCAACAGTTAATCATGAGGTTATCGTGGAAGACTGCATTGAATGGACcggcgg                                                                       >  1:340644/1‑301 (MQ=255)`  
                                                                                                                                                                                                                                      `agtcaccaagtccgttaagaaagtcgttaaggaaggggctcgtccggttaaacaggttgctggcggtctGGCTGGTGGTACTGGTGAAGCACAGATGGTGGAAGTACCACAAGCTGCCGCACAGATTGTTGACGTACCTGAGAAAGAGGTTTCCACTGAGGACGAAGCACAGACAGAAAGCGGACGCAAGAAAGCTCGTGCTGGCGGTAAGAAATCCTTGAGTGTAGCCCGTAGCTCCGGTGGCGGTATCAACATTTAATCAGGAGGTTATCGTGGAAGACTGCATTGAATGGACCGGAgg                                                                       <  1:220937‑M2/232‑1 (MQ=255)`  
                                                                                                                                                                                                                                            `gaaGAAAGTCACCAAGGAAGGGGCTCGTCCGGTTAAACAGGTTGCTGGCGGTCTAGCTGGTCTGGCTGGTGGTACTGGTGAAGCACAGATGGTGGAAGTACCACAAGCTGCCGCACAGATTGTTGACGTACCTGAGAAAGAGGTTTCCACTGAGGACGAAGCACAGACAGAAAGCGGACGCAAGAAAGCTCGTGCTGGCGGTAAGAAATCCTTGAGTGTAGCCCGTAGCTCCGGTGGCGGTATCAACATTTAATCAGGAGGTTATCGTGGAAGACTGCATTGAATGGACCGGAGGTGTCaa                                                                 >  1:214847/2‑301 (MQ=255)`  
                                                                                                                                                                                                                                             `aaGAAAGTCGTTAAGGAAGGGGCTCGGCCGGTTAAACAGGTTTCTGGCGGTCTAGCTGGTCTGGCTGGTGGTACTGGTGAAGCACAGATGGTGGAAGTACCACAAGCTGCCGCACAGATTGTTGACGTACCTGAGAAAGAGGTTTCCACTGAGGACGAAGCACAGACAGAAAGCGGACGCAAGAAAGCTCGTGCTGGCGGTAAGAAATCCTTGAGTGTAGCCCGTAGCTCCGGTGGCGGTATCAACATTTAATCAGGAGGTTATCGTGGAAGACTGCATTGAATGGACCGGAGGTGTCAAc                                                                <  1:132352/301‑1 (MQ=255)`  
                                                                                                                                                                                                                                              `aGAAAGTCGTTAAGGAAGGGGCTCGTCCGGTTAAACAGGTTTCTGGCGGTCTAGCTGGTCTGGCTGGTGGTACTGGTGAAGCACAGATGGTGGAAGTACCACAAGCTGCCGCACAGATTGTTGACGTACCTGAGAAAAAGGTTTCCACTGAGGACGAAGCACAGACAGAAAGCGGACGCAAGAAAGCTCGTGCTGGCGGTAAGAAATCCTTGAGTGTAGCCCGTAGCTCCGGTGGCGGTATCAACATTTAATCAGGAGGTTATCGTGGAAGACTGCATTGAATGGAACGGAggctgtctct                                                               >  1:137523/1‑293 (MQ=255)`  
                                                                                                                                                                                                                                              `aGAAAGTCGTTAAGGAAGGGGCTCGTCCGGTTAAACAGGTTTCTGGCGGTCTAGCTGGTCTGGCTGGTGGTACTGGTGAAGCACAGATGGTGGAAGTACCACAAGCTGCCGAACAGATTGTTGACGTACCTGAGAAAGAGGTTTCCACTGAGGACGAAGCACAGACAGAAAGCGGACGCAAGAAAGCTCGTGCTGGCGGTAAGAAATCCTTGATTGTAGCCCGTAGCTCCGGTGGCGGTATCAACATTTAATCAGGAGGTTATCGTGGAAGACTGCATTGAATGGACCGGAGGTGTCAAct                                                               <  1:323284/301‑1 (MQ=255)`  
                                                                                                                                                                                                                                               `gAAAGTCGTTAAGGAAGGGGCTCGTCCGGTTAAACAGGCTGCTGGCGGTCTAGCTGGTCTTGCTGGTGGTACTGGTGAAGCACAGATGGTGGAAGTACCACAAGCTGCCGCACAGATTGTTGACGTACCTTAGAAAGAGGTTTCCACTGAGGACGAAGCACAGACAGAAAGCGGACGCAAGAAAGCTCGTGCTGGCGGTAAGAAATCCTTGAGTGTAGCCCGTAGCTCCGGTGGCGGTATCAACATTTAATCAGGAGGTTATCGTGGAAGACTGCATTGAATGGACCGTAGGTGTCAActc                                                              <  1:59448/301‑1 (MQ=255)`  
                                                                                                                                                                                                                                                 `ccgttaagaaagtcgttatggaaggggctcgtccggttaaacaggttgctggcggtctGGCTGGTGGTACTGGTGAAGCACAGATGGTGGAAGTACCACAAGCTGCCGCACAGATTGTTGACGTACCTGAGAAAGAGGTTTCCACTGAGGACGAAGCACAGACAGAAAGCGGACGCAAGAAAGCTCGTGCTGGCGGTAAGAAATCCTTGAGTGTAGCCCGTAGCTCCGGTGGCTGTATCAACATTTAATCAGGAGGTTATCGTGGAAGACTGCATTGAATGGACCGGAGGTGTCAAATCTa                                                            >  1:281066‑M2/59‑301 (MQ=255)`  
                                                                                                                                                                                                                                                      `gTTAAGGAAGGGGCTCGTCCGGTTAAACAGGTTTCTGGCGGTCTAGCTGGTCTGGCTGGTGGTACTGGTGAAGCACAGATGGTGGAAGTACCACAAGCTGCCGCACAGATTGTTGACGTACCTGAGAAAGAGGTTTCCACTGAGGACGAAGCACAGACAGAAAGCTGACGCAAGAAAGCTCGTGCTGGCGGTAAGAAATCCTTGAGTGTAGCCCGTAGCTCCGGTGGCGGTATCAACATTTAATCAGGAGGTTATCGTGGAAGACTGCATTGAATGGACCGGAGGTGTCAACTCTAAgggc                                                       >  1:211861/1‑300 (MQ=255)`  
                                                                                                                                                                                                                                                       `agaaagtcgttaaggaaggggctcgtccggttaaacaggttgctggcggtctGGCTGGTGGTACTGGTGAAGCACAGATGGTGGAAGTACCACAAGCTGCCGCACAGATTGTTGACGTACCTGAGAAAGAGGTTTCCACTGAGGACGAAGCACAGACAGAAAGCGGACGCAAGAAAGCTCGTGCTGGCGGTAAGAAATCCTTGAGTGTAGCCCGTAGCTCCGGTGGCGGTATCAACATTTAATCAGGAGGTTATCGTGGAAGACTGCATTGAATGGACCGGAGGTGTCAACTCTAAGGGtt                                                      <  1:302751‑M2/249‑1 (MQ=255)`  
                                                                                                                                                                                                                                                           `ggaaggGGCTCGTCCGGTTAAACAGGTTTCTGGCGGTCTAGCTGGTCTGGCTGGTGGTACTGGTGAAGCACAGATGGTGGAAGTACCACAAGCTGCCGCACAGATTGTTGACGTACCTGAGAAAGAGGTTTCCACTGAGGACGAAGCACAGACAGAAAGCGGACGCAAGAAAGCTCGTGCTGGCGGTAAGAAATCCTTGAGTGTAGCCAGTAGCTCCGGTGGCGGTATCAACATTTAATAAGGAGGTTATCGTGGAAGACTGCATTGAATGGACCGGAGGTGTCAACTCTAAGGGTTATgg                                                  <  1:306682/301‑1 (MQ=255)`  
                                                                                                                                                                                                                                                           `gctcggagatgtgtataagagagaggttaaacaggttgctggcggtctGGCTGGTGGTACTGGTGAAGCACAGATGGTGGAAGTACCACAAGCTGCCGCACAGAATGTTGACGTACCTGAGAAAGAGGTTTCCACTGAGGACGAAGCACATACAGAAAGCGGACGCAAGAAAGCTCGTGCTGGCGGTAAGAAATCCTTGAGTGTAGCCCGTAGCTCCGGTGGCTGTATCAACATTTAATTAGGAGGTTATCGTGGAAGACTGCATTGAATGGACCGTAGGTGTCAACTCTAAGGGTTATgg                                                  <  1:312415‑M2/253‑1 (MQ=255)`  
                                                                                                                                                                                                                                                            `gtcgttaaggaaggggctcgtccggttaaacaggttgctggcggtctGGCTGGTGGTACTGGTGAAGCACAGATGGTGGAAGTACCACAAGCTGCCGCACAGATTGTTGACGTACCTGAGAAAGAGGTTTCCACTGAGGACGAAGCACAGACAGAAAGCGGACGCAAGAAAGCTCGTGCTGGCGGTAAGAAATCCTTGAGTGTAGCCCGTAGCTCCGGTGGCGGTATAAACATTTAATCAGGAGGTTATAGTGGAAGACTGCATTGAATGGACCGGAGGTGTCAAATCTAAGGGGTAtggg                                                 >  1:185375‑M2/48‑300 (MQ=255)`  
                                                                                                                                                                                                                                                            `ggcgttaaggaaggggctcgtccggttaaacaggttgctggcggtctGGATGGTGGTACTGGTGAAGCACAGATGGTGGAAGTACCACAAGCTGCCGCACAGATTGTTGACGTACCTGAGAAAGAGGTTTCCACTGAGGACGAAGCACAGACAGAAAGCGGACGCAAGAAAGCTCGTGCTGGCGGTAAGAAATCCTTGAGTGTAGCCCGTAGCTCCGGTGGAGGTATCAACATTTAATCAGGAGGTTATCGTGGAAGACTGCATTGAATGGACCGGAGGTGTCAACTCTAAGGGTTATGGt                                                 <  1:193038‑M2/254‑1 (MQ=255)`  
                                                                                                                                                                                                                                                                  `aaggaatgggctcgtccggttaaacaggttgctggcggtctGGCTGGTGGTACTGGTGAAGCACAGATGGTGGAAGTACCACAAGCTGCCGCACAGATTGTTGACGTACCTGAGAAAGAGGTTTCCACTGAGGACGAAGCACAGACAGAAAGCTGACGCAAGAAAGCTCGTGCTGGCGGTAAGAAATCCTTGAGTGTAGCCCGTAGCTCCGGTGGCGGTATCAACATTTAATCAGGAGGTTATCGTGGAAGACTGCATTGAATGGACCGGAGGTGTCAACTCTAAGGGTTATGGTCGTAAg                                           <  1:338725‑M2/260‑1 (MQ=255)`  
                                                                                                                                                                                                                                                                   `aggaaggggctcgtccggttaaacaggttgctggctgtctGGCTGGTGGTACTGGTGAAGCACAGATGGTGGAAGTACCACAAGCTGCCGCACAGATTGTGGACGTACCTGAGAAAGAGGTTTCCACTGAGGACGAATCACAGACAGAAAGCGGACGCAAGAAAGCTCGTGCTGGCGGTAATAAATCCTTGAGTGTAGCCCGTAGCTCCGGTGGCGGTATCAACATTTACTCAGGAGGTTATCGTGGAAGTCTGCATTGAATGGACCGGAGGTGTCAACTCTAAGGGTTATGGTCGTAAGt                                          <  1:182336‑M2/261‑1 (MQ=255)`  
                                                                                                                                                                                                                                                                       `aggtgctcgtccggttaaccaggttgctggcggtttGGCTGGTGGTACTGGTGAAGCACAGATGGTGGAAGTACCACAAGCTGCCGCACAGATTGTTGACGTACCTGAGAAAGAGGTTTCCACTGAGGACGAAGCACAGACAGAAAGCGGAAGCAAGAAAGCTCGTGCTGGCGGTAAGAAATCCTTGAGTGTAGCCCGTAGCTCCGGTGGCGGTATCAACATTTAATCAGGAGGTTATCGTGGAAGACTGCATTGAATGGACCGGAGGTGTCAACTCTAAGGGTTATGGTCGTAAGTGGGt                                      <  1:24295‑M2/265‑1 (MQ=255)`  
                                                                                                                                                                                                                                                                                   `ggttaaacaggttgctggcggtctGGCTGGTGGTACTGGTGAAGCACAGATGGTGGAAGTACCACAAGCTGCCGCACAGATTGTTGACGTACCTGAGAAAGAGGTTTCCACTGAGGACGAAGCACAGACAGAAAGCGGACGCAAGAAAGCTCGTGCTGGCGGTAAGAAATCCTTGAGTGTAGCCCGTAGCTCCGGTGGCGGTATCAACATTTAATCAGGAGGTTATCGTGGAAGACTGCATTGAATGGACCGGAGGTGTCAACTCTAAGGGTTATGGTCGTAAGTGGGTTAATGGTAAACt                          <  1:164605‑M2/277‑1 (MQ=255)`  
                                                                                                                                                                                                                                                                                   `ggttaaacaggttgctggcggtctGGCTGGTGGTACTGGTGAAGCACAGATGGTGGAAGTACCACAAGCTGCCGCACAGATTGTTGACGTACCTGAGAAAGAGGTTTCCACTGAGGACGAAGCACAGACAGAAAGCGAACGCAAGAAAGCTCGTGCTGGCTGTAAGAAATCCTTGAGTGTAGCCCGTAGCTCCGGTTGCGGTAGCAACATTTAATCAGGAGGCTATCGTGGAAGACTGCATTGAATGGACCGGGAGTAGCCACTCTAAGGGTTATGGGCGTACGTGGGGTACTGGTAAACt                          >  1:145238‑M2/25‑301 (MQ=255)`  
                                                                                                                                                                                                                                                                                      `taaacaggttgctggcggtctGGCTGGTGGTACTGGTGAAGCACAGATGGTGGAAGTACCACAAGCTGCCGCACAGATTGTTGACGTACCTTAGAAAGAGGTTTCCACTGAGGACGAATCACAGACATATCTCGGACGCAACTAAGCTCGTGCTGGCGGTATGAAATCCTTGTGTGTAGCCCGTAGCTCCGGTGGCGGTCTCAACATTTAATCAGGAGGTTATCGTGGAAGACTGCATTGAATGTACCGGAGGTGTCAACTCTAAGGGTTATGGTCGTAAGTGGGTTAATGGTAAcctttt                       >  1:100102‑M2/22‑299 (MQ=255)`  
                                                                                                                                                                                                                                                                                         `gagggttgggggggggttGGATGGTGGGACTGGTGAAGCAAAGATGGTGGAAGTACCACAAGCTGGCGCACAGATTGTTGACGTACATGAGAAAGAGGTTTCAACTGAGGACGAAGCACAGACAGAAAGCGGAAGCAAGAAAGCTCGTGCTGGCGGTAAGAAATCCTTGAGTGTAGCCCGTAGCTCCGGTGGCGGTATAAACATTTAAGCAGGAGGTTATCGTGGAAGAATGCATTGAATGGACCGGAGGTGTCAACTCTAAGGGTTATGGTCGTAAGTGGGTTAATGGTAAACTTGTGAc                    <  1:175128‑M2/283‑1 (MQ=255)`  
                                                                                                                                                                                                                                                                                                   `ggcggtctGGCTGGTGGTACTGGTGAAGCACAGATGGTGGAAGTACCACAAGCTGCCGCACAGATTGTTGACGTACCTGAGAAAGAGGTTTCCACTGAGGACGAAGCACAGACAGAAAGCGGACGCAAGAAAGCTCGTGCTGGCGGTAAGAAATCCTTGAGTGTAGCCCGTAGCTCCGGTGGCGGTATCAACATTTAATCAGGAGGTTATCGTGGAAGACTGCATTGAATGGACCGGAGGTGTAAACTCTAAGGGTTATGGTCGTAAGTGGGTTAATGGTAAACTTGTGACTCCACATAgg          <  1:197587‑M2/293‑1 (MQ=255)`  

                                                                                                                                                                                                                                                                                                           `|`                                                                                                                                                                                                                                                                                                              
`ATTCACCACAAAGGAGACATTCTGGATGTAACGTCCGTTGTCATTAATCCTGAGGCAGACTCTAAGGGCTTACAGCGATTCCTAGCGAAACGCTTTAAGTACCTTGCGGAACTCCACGATTGCGATTGGGTGTCTCGTTGTAAGCATGAAGGCGAGACAATGCGTGTATACTTTAAGGAGGTATAAGTTATGGGTAAGAAAGTTAAGAAGGCCGTGAAGAAAGTCACCAAGTCCGTTAAGAAAGTCGTTAAGGAAGGGGCTCGTCCGGTTAAACAGGTTGCTGGCGGTCTAGCTGGTCTGGCTGGTGGTACTGGTGAAGCACAGATGGTGGAAGTACCACAAGCTGCCGCACAGATTGTTGACGTACCTGAGAAAGAGGTTTCCACTGAGGACGAAGCACAGACAGAAAGCGGACGCAAGAAAGCTCGTGCTGGCGGTAAGAAATCCTTGAGTGTAGCCCGTAGCTCCGGTGGCGGTATCAACATTTAATCAGGAGGTTATCGTGGAAGACTGCATTGAATGGACCGGAGGTGTCAACTCTAAGGGTTATGGTCGTAAGTGGGTTAATGGTAAACTTGTGACTCCACATAGGCACATCTA  >  NC_001604.1/19346‑19945`

  

Alignment Legend

Aligned base mismatch/match (shaded by quality score): `ATCG/ATCG` `< 3 ≤` `ATCG/ATCG` `< 10 ≤` `ATCG/ATCG` `< 23 ≤` `ATCG/ATCG` `< 34 ≤` `ATCG/ATCG` `< 38 ≤` `ATCG/ATCG`

Unaligned base: `atcg`    Masked matching base: `atcg`    Alignment gap: `‑`    Deleted base: `‑`

Reads not counted as support for junction

read\_name Not counted due to insufficient overlap past the breakpoint.

read\_name Not counted due to not crossing MOB target site duplication.
